# Kinetics of Inclusion Body Formation and Its Correlation with the Characteristics of Protein Aggregates in *Escherichia coli*

**DOI:** 10.1371/journal.pone.0033951

**Published:** 2012-03-29

**Authors:** Arun K. Upadhyay, Aruna Murmu, Anupam Singh, Amulya K. Panda

**Affiliations:** Product Development Cell, National Institute of Immunology, Aruna Asaf Ali Marg, New Delhi, India; Baylor College of Medicine, United States of America

## Abstract

The objective of the research was to understand the structural determinants governing protein aggregation into inclusion bodies during expression of recombinant proteins in *Escherichia coli*. Recombinant human growth hormone (hGH) and asparaginase were expressed as inclusion bodies in *E.coli* and the kinetics of aggregate formation was analyzed in details. Asparaginase inclusion bodies were of smaller size (200 nm) and the size of the aggregates did not increase with induction time. In contrast, the seeding and growth behavior of hGH inclusion bodies were found to be sequential, kinetically stable and the aggregate size increased from 200 to 800 nm with induction time. Human growth hormone inclusion bodies showed higher resistance to denaturants and proteinase K degradation in comparison to those of asparaginase inclusion bodies. Asparaginase inclusion bodies were completely solubilized at 2–3 M urea concentration and could be refolded into active protein, whereas 7 M urea was required for complete solubilization of hGH inclusion bodies. Both hGH and asparaginase inclusion bodies showed binding with amyloid specific dyes. In spite of its low β-sheet content, binding with dyes was more prominent in case of hGH inclusion bodies than that of asparaginase. Arrangements of protein molecules present in the surface as well as in the core of inclusion bodies were similar. Hydrophobic interactions between partially folded amphiphillic and hydrophobic alpha-helices were found to be one of the main determinants of hGH inclusion body formation. Aggregation behavior of the protein molecules decides the nature and properties of inclusion bodies.

## Introduction

Inclusion bodies (IBs) are formed during high level expression of heterologous proteins in *E. coli* [1], [2]. These are often localized in cytoplasm or periplasm of the expression hosts and seen as dense refractile aggregates under electron microscope [3], [4]. Aggregation of proteins during high level expression is generally attributed to high local concentration of naive polypeptide chains emerging from ribosomes, inefficient folding and unavailability of chaperones [5], [6]. These factors lead to the formation of partially folded or misfolded protein intermediates in cytoplasm. Usually, these intermediates have surface exposed hydrophobic patches which come together to form large, amorphous inclusion body (IB) aggregates [7]. Isolation of bioactive protein from inclusion bodies is cumbersome and needs understanding of protein aggregation behavior [8], [9]. Although IBs have been characterized as amorphous aggregates, they are not merely a cluster of disordered folding intermediates [10], [11]. Rather, inclusion body proteins are often enriched in β-sheet structures [12]–[15] and bind to amyloid specific dyes like Thioflavin-T and Congo-Red [14], [16]–[18]. Moreover, inclusion bodies have ability to seed aggregation of structurally similar proteins, a characteristic property of amyloids [14]. Aggregation leading to inclusion body formation has been reported to be very specific in nature as they mostly consist of the expressed recombinant proteins [16], [19]. Size distribution of IB aggregates, as well as their other physical and structural properties depends on cultivation and induction conditions [20], [21]. Reversibility of inclusion body formation [22] and existence of native-like structure of proteins in inclusion bodies has also been reported [12]–[13], [23]–[24]. Besides this, there are reports suggesting the presence of biologically active protein molecules in inclusion bodies [25]–[28]. Inclusion body aggregates formed at lower temperature have fully functional bioactive proteins and are called as non-classical inclusion bodies whereas tough, protease resistant aggregates are called as classical inclusion bodies [29], [30]. However, inclusion bodies produced at higher temperature has been reported to have functional protein in them [31]. The formation of insoluble aggregates of recombinant proteins during high level expression is driven by the association of partially folded or misfolded intermediates. These aggregates have been thought to be resistant to proteases and solubilized only in high molar concentration of chaotropes. It has also been reported that inclusion body aggregates can be solubilized using mild solubilization agent without disturbing the native-like secondary structures of proteins [32]–[34].

There are two proposed models describing the formation of inclusion bodies as a consequence of the self assembly of non-native monomers into growing polymers of higher sizes [35]. The first model proposes that inclusion body aggregation proceeds from a single or limited number of nucleation sites by accumulation of misfolded intermediates. As these nucleation aggregates are thermodynamically stable entities, the addition of misfolded monomers on these aggregates are favored. The second model treats IBs as aggregate of aggregates in which small size aggregates tend to associate themselves to give rise to one or more bigger aggregates. In spite of the availability of a large amount of literature on inclusion body formation, the mechanism of inclusion body formation and the nature of intermediates involved in different types of aggregates are still not clear.

In present work, Human growth hormone (hGH) and *E. coli* L-asparaginase II, were cloned and expressed as IBs in *E. coli*. Kinetics of inclusion body formation, their morphological and structural characteristics were studied by transmission electron microscopy (TEM), size distribution analysis, proteolytic degradation, solubilization, Congo Red and Th-T dye binding assays, and FTIR spectroscopy. The objective was to unravel the aggregation behavior of different proteins expressed as inclusion bodies in *E. coli* so that improved method for their solubilization could be developed for high throughput recovery of bioactive protein.

## Results

### Proteinase K degradation profiles of asparaginase and hGH inclusion bodies

**Figure 1:**
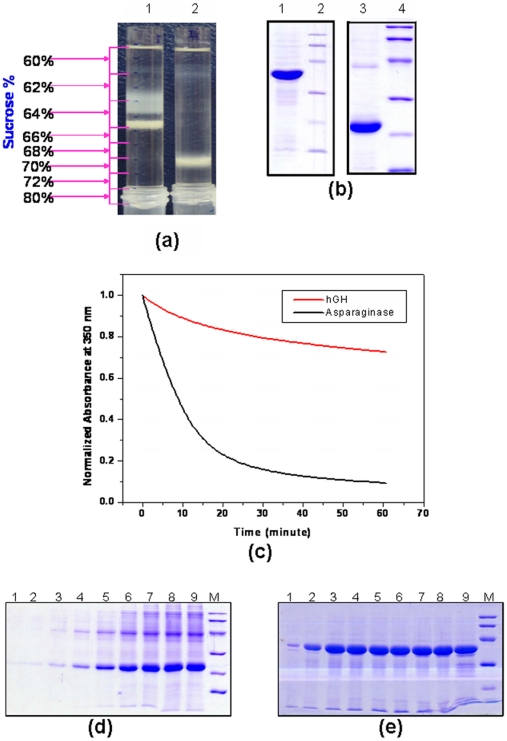
Purification, proteolytic digestion and solubilization profile of human growth hormone and asparaginase inclusion bodies. (a) Purification of inclusion bodies by sucrose density gradient ultracentrifugation, tube one : asparaginase IB, tube two: hGH inclusion bodies (b) SDS-PAGE analysis of purified hGH (21 kDa, lane 1) and asparaginase (37 kDa, lane 3) inclusion bodies. Lane 2 and 4, LMW marker: 97, 66, 45, 30, 20.1 and 14.4 kDa. (**c**) Kinetics of proteolytic digestion of inclusion body aggregates by Proteinase K. SDS-PAGE of solubilized inclusion body supernatants. (**d**) hGH IBs. Lane 1–9, supernatants of 0 to 8 molar urea. (**e**) Asparaginase IBs. Lane 1–9, supernatants of 0 to 8 molar urea; lane M, LMW marker (97, 66, 45, 30, 20.1 and 14.4 kDa).

**Figure 2:**
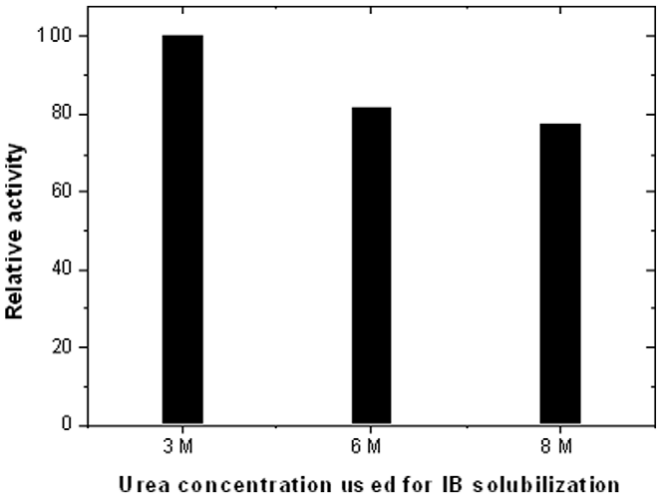
Relative activities of refolded asparaginase after solubilization of asparaginase IBs in different concentrations of urea.

To understand the differential physical properties of different inclusion body aggregates, proteolytic susceptibilities of the recombinant hGH and asparaginase inclusion bodies expressed in *E. coli* were analyzed. Both the proteins were expressed as inclusion bodies and purified by sucrose density gradient ultracentrifugation (Fig. 1a). In spite of its low molecular weight, hGH inclusion bodies were denser (tube 2 in Fig. 1a) in comparison to the asparaginase inclusion bodies (tube 1 in Fig. 1a). Asparaginase and hGH were expressed as 37 kDa and 21 kDa protein respectively as shown in SDS-PAGE (Fig. 1b). The purified inclusion bodies were subjected to proteinase K treatment which indicated differential susceptibilities of these IBs to proteinase K (Fig. 1c). Inclusion bodies of hGH were found to be resistant to proteinase K whereas asparaginase IBs were highly prone to proteinase K attack. In 20 minutes, 80% of asparaginase IBs was degraded whereas only 20% degradation was observed for hGH IBs. This apparently indicated the tough and compact nature of hGH inclusion bodies in comparison to those of asparaginase inclusion bodies. This was further confirmed by the denaturation profiles of hGH (Fig. 1d) and asparaginase IBs (Fig. 1e) at different concentrations of urea. Complete solubilization of hGH IBs occurred at 7 molar urea concentration. However, just 2–3 molar urea was found to be sufficient for complete solubilization of asparaginase IBs. Furthermore, asparaginase IBs solubilized in 3 M urea and refolded by pulsatile dilution method resulted in bioactive protein. The activity was comparable to the asparaginase refolded after solubilization in 6 M or 8 M urea (Fig. 2). This information suggested the soft and delicate nature of asparaginase IBs in comparison to dense and compact nature of hGH inclusion bodies. It can be concluded that hGH inclusion bodies were of classical type whereas asparaginase was expressed as non-classical inclusion bodies.

### Kinetic of inclusion body formation by size analysis of protein aggregates

**Figure 3:**
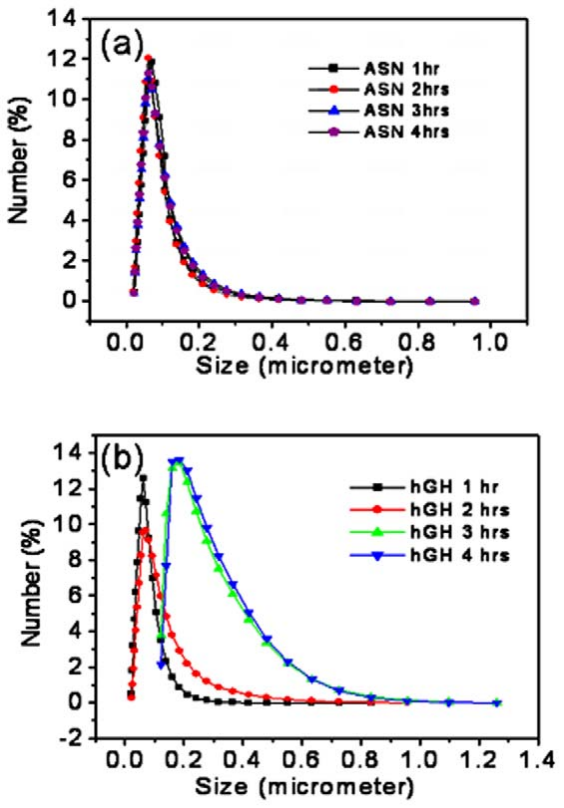
Size distribution patterns of IBs isolated from *E. coli* cells harvested at different time points after induction. (**a**) Asparaginase IBs. (**b**) hGH IBs. Colored bars indicate the harvesting time.

**Figure 4:**
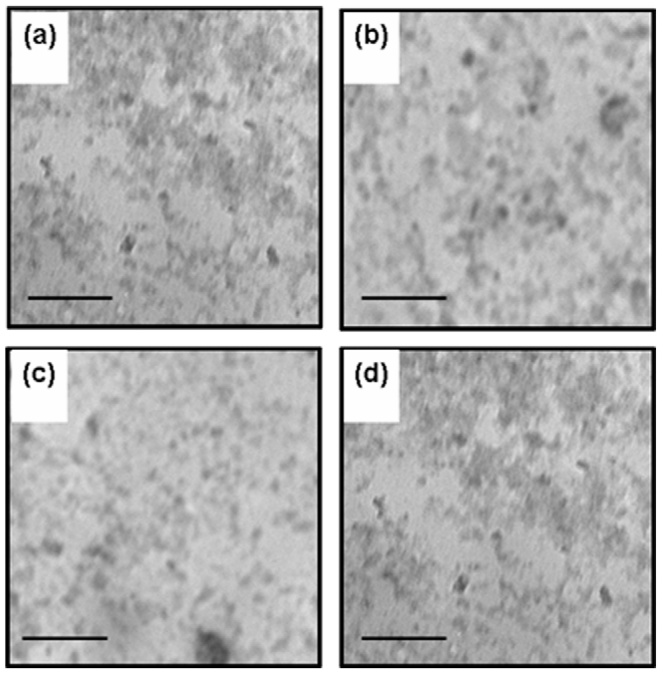
Transmission electron micrograph of purified asparaginase inclusion bodies. (**a**), (**b**), (**c**) and (**d**) asparaginase IBs isolated after 1, 2, 3 and 4 hours of IPTG induction respectively. Bar represents 2 µm.

Kinetics of inclusion body formation with induction time, exact mechanism of nucleation and growth of inclusion body aggregates are not well understood. To understand this, sizes of IBs isolated from *E. coli* cells harvested at different time points (1, 2, 3 and 4 hours duration) after induction were analyzed. Ultra pure IBs from these *E. coli* cells were isolated by procedures as described in materials and methods. Size distribution patterns of hGH and asparaginase IBs analyzed by particle sizer are presented in Figure 3. Size distribution pattern of asparaginase IBs remained same for four hours duration after induction (Fig. 3a). Most of the asparaginase IBs sizes were in range of 100–200 nm sizes. There were no distinct nucleation and growth phases present within the shortest possible time limit of 60 minutes after induction. However the size of hGH inclusion bodies increased progressively and saturated at around 4 hours after induction (Fig. 3b). After 4 hours of induction, most of the hGH inclusion bodies were around 200–800 nm with very less proportion having size less than 100 nm. This was also supported by transmission electron micrographs of these IBs. Size of pure asparaginase IBs isolated after different time points of IPTG induction was almost same (Fig. 4). However, there were approximately 15% aggregates in range of 20–40 nm in inclusion body preparations of different post induction periods. The expression level of asparaginase saturated only after four hours of IPTG induction while inclusion body size saturated within 60 minutes after induction. The size of aggregates could have increased after 60 minutes of induction if newly synthesized polypeptide chains would have been deposited on the same seed in a single cell. But it was contrary to size distribution result obtained for asparaginase IBs. Thus, it can be concluded that multiple nucleation seeds were formed in a single cell of *E. coli* during expression of recombinant asparaginase. The growth of these seeds occurred for short duration and got saturated within 1 hour and further new seeds were formed during protein expression.

**Figure 5:**
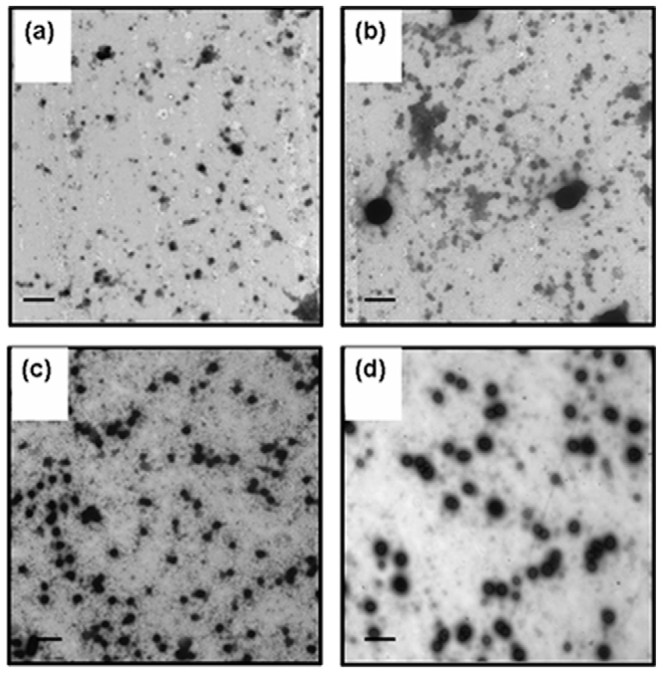
Transmission electron micrograph of purified hGH inclusion bodies. (**a**), (**b**), (**c**) and (**d**) are hGH IBs isolated after 1, 2, 3 and 4 hours of IPTG induction respectively. Bar represents 2 µm.

In contrast, size distribution pattern of hGH inclusion bodies formed after induction for different durations, indicated the presence of distinct nucleation and growth phases during IB formation (Fig. 3b). The maximum size of IBs after 4 hours of IPTG induction was in range of 200 to 800 nm. The seeding of aggregation started immediately after induction and there was gradual increase in size of IB aggregates till 3 hours of post induction period (Fig. 3b). The increase in size of IB aggregates was highest between 2–3 hours of post induction period. After one hour of induction, 90% of IB aggregates were of smaller size (25–150 nm). With increase in post induction period, the population of these smaller size IBs was reduced to 65% at 2 hours and at the end of 3 hours after induction, just 10% IB aggregates were found to be smaller than 150 nm. This indicated that hGH IB formation starts immediately after induction, aggregation seeds are formed and they grow in size continuously during expression period. Increase in size of hGH inclusion bodies at different time points after induction was also confirmed by TEM analyses (Fig. 5).

Thus, it can be concluded that different proteins follow different aggregation mechanisms during recombinant protein expression in *E. coli*. Expression of asparaginase in *E. coli* leads to the formation of large number of aggresomes (small aggregates) in a single cell which loosely associate amongst themselves to form bigger aggregates. While expression of hGH IBs in *E. coli* results in the formation of single large aggregate in a cell by continuous deposition of protein molecules on a nucleation seed. The proteolytic digestion and solubilization profile of hGH and asparaginase IBs were in agreement with size distribution pattern. Tough and large IBs are formed by sequential deposition whereas soft and small IB aggregates are formed immediately after IPTG induction.

### Solubilization and proteolytic susceptibilities of inclusion bodies harvested after different induction time

**Figure 6:**
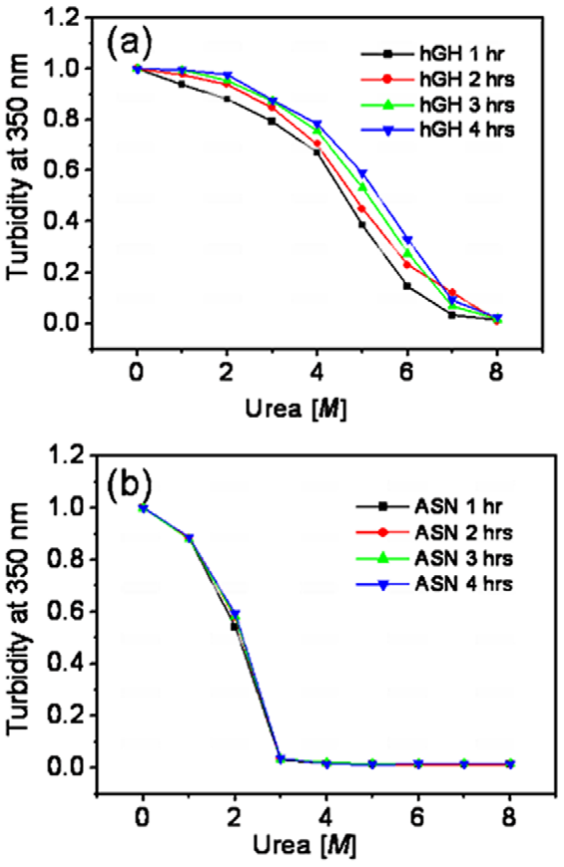
Solubilization profiles of inclusion bodies isolated from cell harvested at different time points after induction. (**a**) hGH IBs (**b**) Asparaginase IBs. Colored bars indicate the harvesting time.

To measure the extent of compactness of protein aggregates in inclusion bodies, purified IBs were subjected to different denaturing conditions and proteinase K digestion. Solubilization profile of hGH and asparaginase inclusion bodies in different molar concentrations of urea was determined by measuring the turbidity of solubilized samples at 350 nm (Fig. 6). It was observed that hGH inclusion bodies were progressively more soluble with increasing urea concentration from 1–8 M (Fig. 6a). Complete solubilization of inclusion body aggregates occurred at 7 M urea solution. Interestingly, the solubility of proteins from inclusion bodies isolated at early hours after induction was more in comparison to those isolated at 2–4 hours after induction (Fig. 6a). However smaller sized inclusion bodies of hGH isolated after early hour of induction were not soluble in low concentration of urea as observed for asparaginase. In contrast, asparaginase inclusion bodies were completely soluble in 2–3 molar urea concentration (Fig. 6b). For asparaginase, solubilization pattern of IB proteins in different molar concentrations of urea was independent of harvest time after IPTG induction (Fig. 6b).

**Figure 7:**
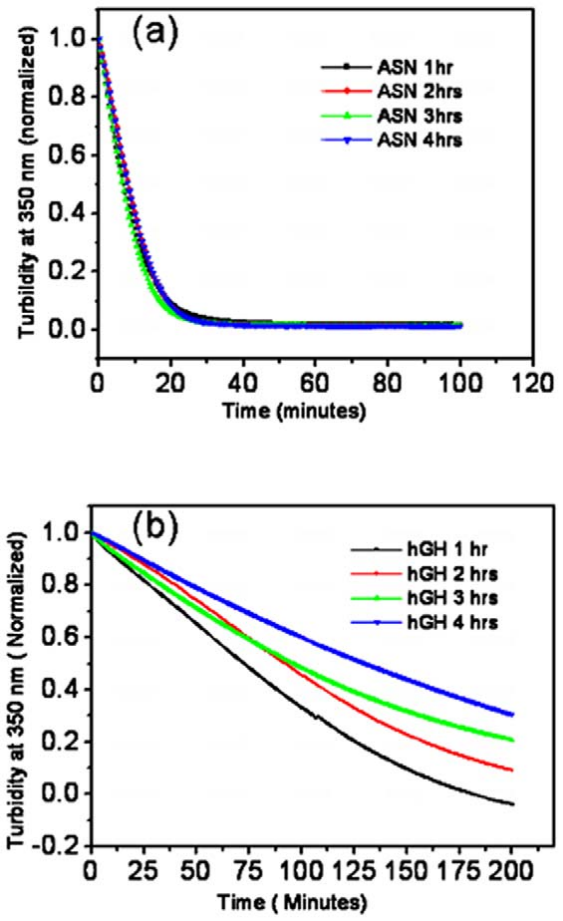
Proteinase K degradation profiles of inclusion bodies isolated from *E. coli* cells harvested at different time points after induction. (**a**) Asparaginase IBs. (**b**) hGH IBs. Colored bars indicate the harvesting time.

This difference in solubilization profile of hGH inclusion bodies isolated from cells harvested at different time points after induction could be due to differences in composition of IB aggregates. The smaller inclusion body aggregates (isolated after 1 hour of induction) got solubilized at slightly lower concentration of urea owing to their large accessible surface area. However, this possibility can be negated as sufficient time was given for solubilization of these IBs. Thus, it can be inferred that increase in post induction period makes hGH inclusion bodies more resistant to denaturants. Similar results were obtained from proteinase K degradation study (Fig. 7). Asparaginase inclusion bodies were found to be very prone to proteinase K attack and there was no effect of post induction time on susceptibility of these inclusion bodies to proteinase K degradation (Fig. 7a). Asparaginase inclusion bodies showed steep slope for denaturant based solubilization and proteinase K digestion. Proteinase K digestion required less time to degrade asparaginase inclusion bodies. This inferred that the stabilizing forces between aggregating species in asparaginase inclusion bodies are weak and unstable. Removal of molecules from the asparaginase IB surface during solubilization was rapid and occurred within a narrow range of urea concentration. However, hGH inclusion bodies isolated after 1 hour of induction were more prone to proteinase K degradation in comparison to inclusion bodies isolated after 2, 3 and 4 hours of post induction (Fig. 7b). There was gradual decrease in susceptibility of human growth hormone IBs to proteinase K with increase in post induction time. Human growth hormone inclusion bodies were more compact and required higher concentration of denaturant for complete solubilization. The concentrations of urea for 50% solubilization of hGH inclusion bodies isolated at 1, 2, 3, and 4 hours of post induction were found to be 4.7 M, 4.95 M, 5.2 M and 5.5 M respectively. This showed that the compactness and strength of interaction between aggregating species in hGH inclusion bodies increased with increase in post induction time. No such changes were observed in case of asparaginase inclusion bodies. Solubilization and proteolytic data inferred that not only the size but also the quality of asparaginase inclusion body aggregates do not change with increase in post induction time.

**Figure 8:**
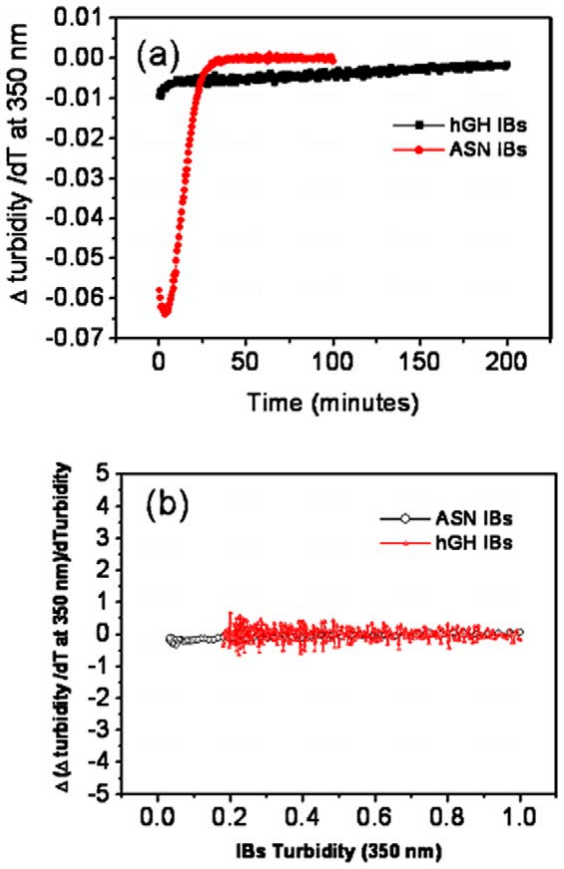
Proteinase K degradation profiles of hGH and asparaginase inclusion bodies isolated after 4 hours of induction. (**a**) Rate of degradation of IBs with time. (**b**) Rate of change in degradation rate of IBs with decrease in turbidities.

Homogeneity in arrangement of protein molecules in inclusion bodies was deduced by calculating the change in rate of proteolytic degradation of IBs with time. It was of great interest to know whether there is any difference between the arrangement of protein molecules present in the core and the peripheral regions of IB aggregates. Proteinase K degradation pattern of IBs isolated after four hours of induction was analyzed. The first derivative graph of proteinase K digestion of IBs revealed that the rate of degradation decreased with reaction time (Fig. 8a). This could be either due to the compact nature of inner layers of IBs which get exposed after removal of upper layers or this may be due to decreased IB concentration (as it acts as a substrate for proteinase K, continuous conversion of inclusion body aggregates into degraded fragments during reaction will decrease the substrate concentration, thus reaction rate). To find out the possible reason for this degradation pattern, rate of change in degradation rate was plotted with respect to turbidity (i.e. substrate concentration) of inclusion bodies measured at 350 nm at different time points of reaction (Fig. 8b). The rate of change of degradation rate was negligible with respect to substrate concentration. It can only be possible in an enzyme catalyzed reaction if substrates (IBs) are similar in nature. Thus, it can be concluded that smaller aggregates which form during proteinase K degradation have similar morphology and topology as intact IBs and there is no difference in arrangement of protein molecules present in core and peripheral regions of both asparaginase and hGH inclusion bodies.

### Binding of amyloid specific dyes to inclusion bodies

**Figure 9:**
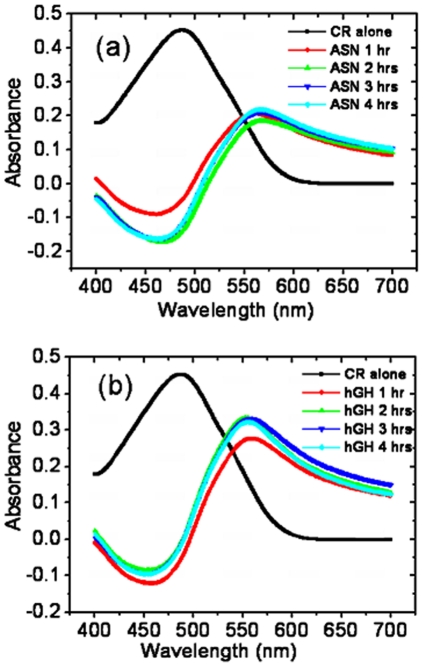
Spectral characteristics of Congo-Red (CR) and difference spectra of IBs. (**a**) Asparaginase and (**b**) hGH inclusion bodies isolated at different time points after induction. Concentrations of CR and IBs used for assay were 10 µM and 25 µg/ml respectively.

It has been observed that amyloid fibrils interact specifically with Congo red (CR) dye which depends on secondary structure of interacting entities of fibril, especially β-pleated sheet conformation [36]. The cross β-sheet conformation of amyloids appears to be the crucial factor in CR binding. Other proteins that lack or contain only minor proportion of β-sheet structure do not stain with CR [37]–[38]. The interaction of amyloids with CR also induces shift in the characteristic spectrum of CR which depends on the nature of aggregate conformation. Inclusion body aggregates consist of partially ordered secondary structures and there are reports indicating the binding of IBs with CR dye [14], [16]–[18]. Therefore, binding of IBs to CR was analyzed to study their amyloidogenic nature. CR alone exhibits absorption maximum at 490 nm, which shifts to higher wavelength once it binds to amyloid material. Asparaginase and hGH IBs isolated at different time points after induction promoted a strong red shift in the absorption maxima (Fig. 9). The difference spectra showed broad peaks at 565 nm for asparaginase IBs and at 560 nm for hGH IBs. However, absorbance of CR with IBs isolated at early time point of post induction was less in comparison to that of inclusion bodies harvested after later time points. The absorbance of CR bound hGH IBs was higher than that of asparaginase IBs indicating that hGH IBs are more amyloidogenic in nature in comparison to asparaginase IBs.

**Figure 10:**
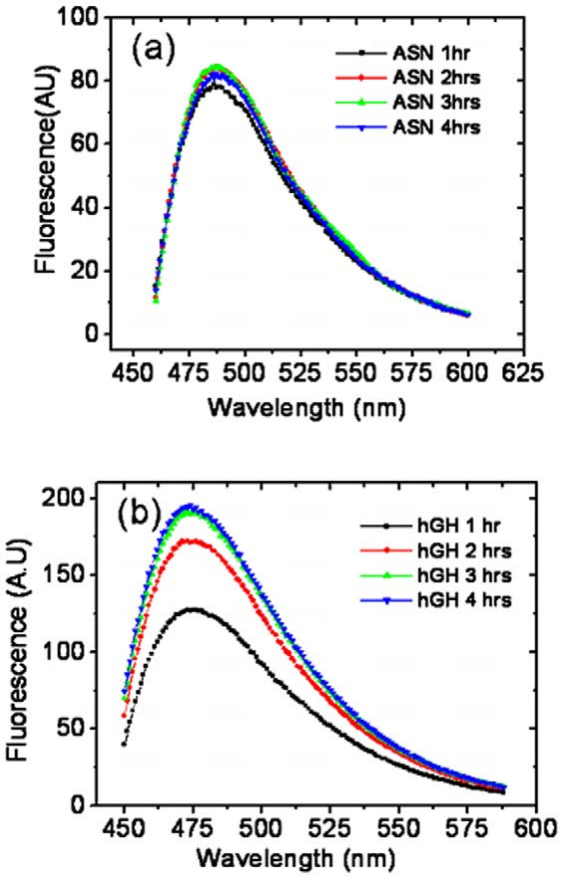
Spectral characteristics of Th-T binding with IBs. (**a**) Asparaginase and (**b**) hGH inclusion bodies isolated at different time points after induction. Concentrations of Th-T and IBs used for assay were 75 µM and 50 µg/ml respectively.

These results were also supported by thioflavin-T (Th-T) binding, which is an amyloid specific fluorescent dye. Thioflavin-T also undergoes characteristic spectral alteration on binding to amyloid aggregates, which does not occur on binding to the precursor polypeptides, monomers, or amorphous aggregates of peptides and proteins [39], [40]. Binding of amyloid fibril with Th-T alters excitation and emission maxima of dye. Emission maxima for hGH and asparaginase IBs were around 475 nm and 485 nm. In presence of amyloid aggregates, it showed excitation maxima at 440 nm and emission maxima at 482 nm. Binding to amyloid aggregates result in large enhancement in fluorescence intensity of Th-T. The binding of Th-T dye with hGH IBs brought sharp increase in fluorescence intensity in comparison to asparaginase IBs at equal protein concentrations (Fig. 10). It was observed that increase in post induction period resulted in formation of hGH inclusion bodies which showed stronger binding to Th-T dye. Asparaginase inclusion bodies isolated at different time points after induction had similar binding strength with Th-T. The difference in CR and Th-T binding to asparaginase and hGH inclusion bodies could be either due to variation in arrangement of protein molecules inside inclusion body aggregates or due to the types and proportion of secondary structural elements present in IBs.

### Structural analysis of inclusion body proteins by FTIR spectroscopy

**Figure 11:**
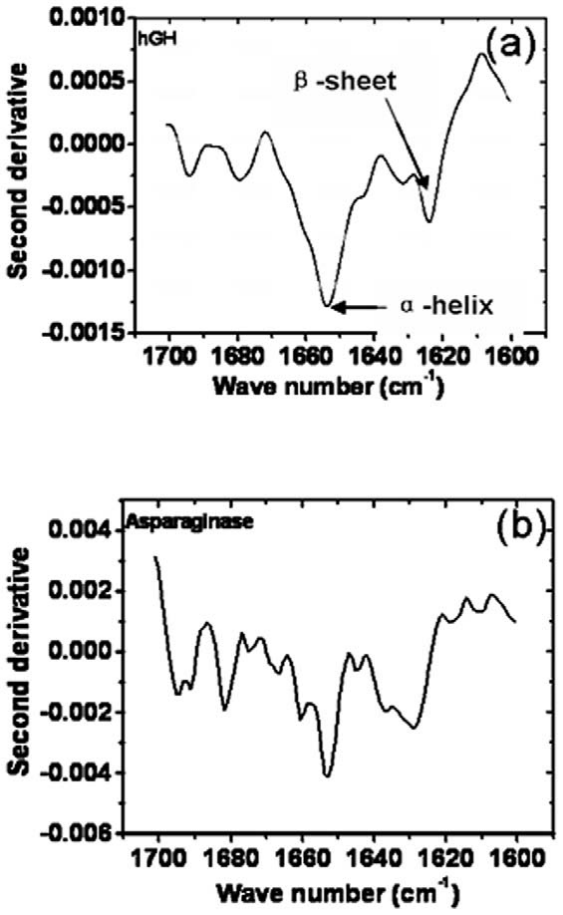
Second derivative FTIR spectra of hGH and asparaginase IBs. (**a**) Second derivative spectrum of hGH IBs. (**b**) Second derivative spectrum of asparaginase IBs in amide band region. Broader peak in 1620–1640 cm-1 and 1680–1685 cm-1 range indicates large beta sheet content.

To find out the possible role of secondary structural elements of hGH and asparaginase IBs in differential binding properties to CR and Th-T dyes, their FTIR spectra were analyzed. Native hGH is predominantly a helical protein with small number of loops and turns, while asparaginase consists of both helices and β-sheets as well as large number of loops and turns. Second derivative spectra of native hGH [41] and hGH inclusion bodies showed that formation of inclusion body aggregates lead to small increase in β-sheet content (Fig. 11a). Second derivative spectra of hGH IBs showed prominent peak area at 1654 cm^−1^, characteristics of α-helices with small peak area at 1623 cm^−1^, characteristic of β-sheet. In spite of small proportions of β-sheet content, hGH inclusion bodies showed very strong binding with CR and Th-T dyes. It was also found to be more resistant to proteolysis and denaturation. From the amino acid sequence of helix forming regions of hGH protein, it was observed that majority of them consist of hydrophobic and amphipathic amino acid residues (Table 1). These residues were present at regular intervals and formed hydrophobic helices. These helices may interact strongly and form very compact and hard aggregates. Moreover, the formation of compact and insoluble aggregates does not necessarily involve the participation of β- sheets and may be formed by strong interaction between amphipathic or hydrophobic helices. FTIR spectra of asparaginase IBs revealed the presence of significantly large proportion of β-sheet content in IBs (Fig. 11b) in comparison to native protein [42]. The crystal structure of asparaginase also entails that the protein is rich in β-pleated secondary structures (Swissprot, P00805, PDBe:3eca). Crystal structure of asparaginase and β-sheet content present in IBs, shown by FTIR spectra (Fig. 11b), are in accordance to its binding properties to amyloid specific dyes. The soft and denaturation prone nature of asparaginase IBs can be due to irregular arrangement of its β-sheet regions, leaving large accessible areas to proteases and denaturants for action.

**Table 1:** Amino acid sequence of hGH (Swiss-Prot, P01241).

>sp|P01241|SOMA_HUMAN Somatotropin
OS = Homo sapiens
MATGSRTSLLLAFGLLCLPWLQEGSAFPTIPL***SRLFDNAMLRAHRLHQLAFDTY***QEFEEAYIPKEQKYSFLQNPQTSLCFSESIPTPSNREETQQSN***LELLRISLLLIQSW***LEPVQFLRSVFANSLVYGAS***DSNVYDLLKDLEEGIQTLMGR***LEDGSPRTGQIFKQTYSKFDTNSHNDDAL***LKNYGLLYCFRKDMDKVETFLRIVQCRS***VEGSCGF

Bold and italics regions indicate presence of hydrophobic residues involves in making hydrophobic and amphipathic helices.

## Discussion

Formation of inclusion body aggregates imposes a severe challenge in purification of bioactive recombinant protein from *E. coli*. The information about structural and physical properties of inclusion bodies during expression conditions is necessary for developing mild solubilization procedures which may result in improved recovery of bioactive protein. Better insight into the structural and physical properties of inclusion bodies can help in developing appropriate solubilization and refolding conditions during purification of bioactive protein from inclusion bodies. The present study reveals that depending on the aggregation properties of proteins, different types of inclusion bodies are formed in *E. coli*. Asparaginase IBs were around 100 nm in size whereas the size of hGH IBs was in the range of 200–600 nm. Small sized inclusion bodies are soft and are soluble in low concentration of urea where as large size inclusion bodies are denser and require high molar concentration of urea for complete solubilization. There was no significant effect of post induction period on quality and size of inclusion body aggregates for asparaginase where as for hGH the inclusion bodies size increased along with induction time. However, hGH inclusion bodies were tough and protease resistant from the beginning. Present study also put forward variation in kinetics of IB formation by different proteins. Proteins which form softer and smaller aggregates showed continuous seeding during expression period as contrary to the denser and harder IB forming proteins. Amyloid specific dye binding assays reveals the amyloidogenic nature of IB aggregates. Also, β-sheet conformation was not found to be solely responsible for protein aggregation into inclusion bodies as observed in the case hGH IBs. It is the interactive forces associated with the secondary structural elements of the protein that govern the aggregation kinetics leading to the formation of different types of IB aggregates.

On the basis of solubility and presence of biological activity, IBs have been categorized as classical and non-classical inclusion bodies [29]. The non-classical inclusion bodies have high amount of correctly folded target protein or its precursor and are produced at lower temperature [30]. Our observations indicated non-classical nature of asparaginase IBs and classical nature of hGH IBs. It can be proposed that non-classical inclusion bodies are less dense, soluble in mild denaturants concentration, susceptible to protease attack, less amyloidogenic and have more defined protein structure in it. Whereas classical inclusion bodies are denser, tough, highly amyloidogenic, more resistant to protease attack, and are only soluble in high molar concentration of chaotropes. The mechanisms of inclusion body formation for recombinant asparaginase and hGH are quite different. The seeding and growth phases of asparaginase inclusion body formation were of shorter duration and resulted in formation of small size (100–200 nm) aggregates in comparison to hGH IBs. In contrast to this, hGH inclusion body formation started from smaller nuclei and grown into bigger size with post induction period. Human growth hormone inclusion bodies were more resistant to proteinase K and showed solubilization profile in urea like a classical inclusion body while asparaginase IBs were more susceptible to denaturants and proteases as contrary to hGH. The post induction period had little effect on proteolytic susceptibility and chaotropic resistivity of asparaginase IBs in comparison to hGH IBs. It showed that increase in post induction period made hGH IBs more resistant to denaturants and proteases. The molecular arrangement of misfolded or native-like polypeptides in IB aggregates are homogenous and uniform from inner core region to peripheral region. Inclusion body aggregates have amyloid like properties and consist of higher percentage of β-sheet conformation in comparison to native proteins. The formation of hGH inclusion bodies during recombinant expression in *E. coli* involves strong interactions between partially folded, amphipathic or hydrophobic α-helices. The study showed that there is fundamental difference in the aggregation behavior, physical and structural characteristics of asparaginase and hGH inclusion bodies. However, the most important information is that non-classical or soft inclusion bodies could be solubilized by low concentration of chaotropes for high throughput recovery of bioactive proteins. Recently, our group has also shown recovery of bioactive hGH after solubilization of hGH inclusion bodies in n-Propanol based mild solubilization agent [43]. These observations suggest the presence of native-like secondary structures in inclusion body aggregates. The variations in size of aggregates of the two proteins may arise due to differences in their nucleation and growth properties during IB formation. However, amino acid sequences and intermittent topologies present in folding intermediates (formed during expression) can also contribute to the nature of inclusion body aggregates.

## Materials and Methods

### Chemicals and Reagents

Culture media ingredients, tryptone and yeast extract were from Difco Laboratories, India. Tris buffer, Glycine, Isoproppyl β-thiogalactopyranoside (IPTG), sodium dodecyl sulphate, PMSF, and deoxy cholic acid were from Amresco, USA. Ammonium persulphate, acrylamide and bis-acrylamide from Sigma chemicals, USA. TEMED, EDTA, Bromophenol blue from BIO-RAD, USA. Coomassie brilliant blue R-250 and ampicillin and kanamycin were from USB Corporation, Cleveland, Ohio. SDS-PAGE low molecular weight marker was purchased from GE Healthcare, USA. Micro BCA assay kit was from Pierce, USA. Urea, proteinase K, Thioflavin-T and Congo Red dyes were from Sigma Chemical Ltd., USA. Glucose, NaCl, reagents and other chemicals were from Qualigen, India. *Escherichia coli* BL21 (DE3) and M 15 strain were from Novagen, USA. Strain DH5α was obtained from Invitrogen, USA. Vectors were obtained from Novagen, USA.

### Cloning, expression and isolation of inclusion bodies

To clone hGH without any tag and its signal sequence, the cDNA fragment coding for hGH was excised with HinfI-HindIII from pRMhGH. This excised cDNA fragment was lacking the first 18 bp. The18 bp were chemically synthesized with HinfI overhang at the 3′-end and NcoI overhang at the 5′-end [5′CATG TTC CCA ACT ATT CCA CTG 3′; 3′AAG GGT TGA TAA GGT GAC TCA5′]. This synthetic oligonucleotide and HinfI-HindIII-digested cDNA fragments were inserted into NcoI-HindIII-digested pQE-60 expression vector (Qiagen,U.S.A.). The construct thus obtained has hGH with just one extra methionine at the N-terminus under the control of the phage T5 promoter. The NcoI site originally present in pQE-60 was lost during construction of this plasmid. *E. coli* M15 cells containing the recombinant expression plasmid (pQE 60-hGH) were grown in LB or complex medium in the presence of kanamycin (25 mg/ml) and ampicillin (50 mg/ml). The cultures were induced with 1 mM IPTG and were further grown for 4 h. Expression of r-hGH in the total cell extracts from both uninduced and induced cultures was checked by SDS-PAGE. L-asparaginase II (ansB) gene was amplified from the genomic DNA of *E. coli* K-12 strain (JM109) using primers (forward) 5′GTG CAG CAC ATA TGT TAC CCA ATA TCA CCA3′ and (reverse) 5′GGC GGG ATC CTT AGT ACT GAT TGA AGA3′. NdeI and BamHI restriction sites were incorporated in the primers to facilitate cloning of the structural asparaginase gene (without its native signal sequence) in the *E. coli* expression vector pET14b in fusion with a six histidine tag at the N-terminus. The resultant recombinant plasmid pET14b-Asp was sequenced to confirm the asparaginase gene insert. *E. coli* BL21 (DE3) cells were transformed with recombinant pET14b plasmid vector to get the expression strain. *E. coli* strain M15 expressing recombinant hGH and BL21 (DE3) expressing recombinant L-asparaginase were grown in LB medium at 37°C for 3.5 hrs. 100 µg/ml ampicillin and 25 µg/ml kanamycin were used in medium in case of hGH expression and 100 µg/ml ampicillin in case of asparaginase expression. Cells were induced during mid log phase of growth with final IPTG concentration of 1 mM. Cells were pelleted at 6000 rpm (rotor SLA-3000, Sorvall Revolution) for 10 minutes at 4°C. Supernatant medium was discarded and one litre culture pellet was resuspended in 20 ml buffer I (50 mM Tris-HCl, 1 mM EDTA, 1 mM PMSF, pH 8.5) containing egg white lysozyme (1 mg/ml) and incubated at room temperature for 2 hours. Resuspended cells were sonicated for 10 cycle (1 min cycle with 1 min gap) using Branson sonifier 450, Germany (probe diameter 13 mm, output voltage 60 and % duty cycle 50). Sonicated cells were centrifuged at 12000 rpm (SA-300 rotor, Sorvall Revolution) for 20 minutes at 4°C. Again, supernatant was discarded and pellet was resuspended in 20 ml buffer I containing 1% Deoxycholic acid (for hGH IBs) and 0.1% Deoxycholic acid (for asparaginase IBs). Pellets were again sonicated and centrifuged as described earlier. Pellet was washed twice with buffer I and final pellet was washed with de-ionized water. Washed pellet was resuspended in 1 ml 50 mM Tris-HCl buffer, pH 8.5.

### Inclusion body purification by sucrose density gradient ultracentrifugation

Isolated IBs were further purified by sucrose density gradient ultra centrifugation. Sucrose step gradient was prepared in ultracentrifuge rotor tubes by drop wise addition of sucrose solution. 1 ml of each of 80%, followed by 72%, 70%, 68%, 66%, 64%, 62%, and 60% (w/v) of sucrose solution was added from bottom to top of the tube to prepare the sucrose gradient. 1 ml inclusion body suspension was added on top of the 60% sucrose layer in the tube, and centrifuged at 1,20,000 g for 6 hours in a swinging rotor at 4°C. Inclusion bodies were seen in the sucrose gradient as a dense layer below impurities. The dense layer was carefully removed by pipetting without disturbing the other layers and washed with Milli-Q water. Purity of the inclusion bodies was checked by 12% SDS-PAGE analysis. Purified inclusion bodies were used for size analysis.

### Solubilization of purified inclusion bodies

Solubilization profile of pure inclusion bodies in urea was determined by measuring turbidity of solubilized samples at 350 nm. Isolated hGH and asparaginase IBs were solubilized in 50 mM Tris-HCl, 5 mM DTT, pH 8.5 buffers containing different molar urea (0–8 molar) and left overnight. After solubilization, turbidities of samples were measured at 350 nm by spectrophotometer (UV 2450, Shimadzu, Japan).

### Asparaginase activity assay

Asparaginase IBs were solubilized in 3 M, 6 M and 8 M urea for 2 hours. Solubilized samples were centrifuged at 13,000 rpm for 20 minutes. Supernatants were then refolded by drop-wise dilution in buffer containing 50 mM Tris-HCl. Asparaginase activity was assayed using method described by Wriston Jr [44]. Briefly, reaction mixture consists of 50 mM Tris-HCl (pH 8.6) and 8.6 mM L-asparagine was incubated at 37°C for 10 minutes. Refolded L-asparaginase enzyme solution (10 µg/ml) was added in reaction mixture and incubated at 37°C. Reaction was stopped by adding 1.5 M trichloro acetic acid at different time points and sample was centrifuged and used for estimation of released ammonia by Nessler's reagent using ammonium sulphate as standard. An international unit (IU) of L- asparaginase is defined as the amount of enzyme required to release one micromole of ammonia per minute under the condition of the assay at saturating substrate concentration.

### Size analysis of inclusion bodies

Asparaginase and hGH inclusion bodies, isolated and purified at different time points after induction of *E. coli* cells, were homogenized and their size distribution was analyzed by Malvern Mastersizer Hydro 2000S (Malvern Instruments, USA). Inclusion body samples were injected into detection chamber with 10% obscurity and were scanned 10 times. Size distributions of inclusion bodies were analyzed by plotting a graph between percentage population and their size of inclusion bodies harvested at different time points after induction. Inclusion bodies isolated after different time of induction were purified and analyzed by transmission electron microscopy. Ultra pure inclusion bodies obtained by sucrose density gradient ultra centrifugation methods were homogenized and diluted to 1 mg/ml. 10 µl of the diluted samples of IBs were placed on carbon-coated copper grids and dried in a desiccator for 30 minutes. Then grids were washed with distilled water and stained with 2% (w/v) uranyl acetate for 5 minutes. Morphology and refractive properties of inclusion bodies were analyzed under transmission electron microscope (Jeol JSM 35CF, Japan).

### Proteolytic digestion of inclusion bodies

Purified hGH and asparaginase inclusion bodies, obtained after different time points of induction, were subjected to proteolytic digestion. Inclusion bodies were diluted to 1 OD at 350 nm in 980 µl of 50 mM Tris-HCl, 150 mM NaCl buffer of pH 8.0. Proteolytic digestion of IBs was initiated by adding 20 µl proteinase K stock (0.2 mg/ml) to the inclusion body solution (at 4 µg/ml final concentration). Proteolytic digestion was monitored for 200 minutes (hGH IBs) and 100 minutes (Asparaginase IBs) by measuring the changes in OD at 350 nm in UV-2450 spectrophotometer (Shimadzu, Japan).

### Amyloid specific assays of inclusion bodies

Asparaginase and hGH IBs were tested for Congo red (CR) binding by the spectroscopic band shift assay [14]. Inclusion bodies were diluted in reaction buffer (10 mM sodium phosphate, pH 7.0 containing 150 mM NaCl) containing 10 µM CR to final protein concentration of 25 µg/ml. Samples were incubated for 10 minutes at room temperature before acquisition of spectra. Absorption spectra were collected together with negative control solutions of dye in the absence of protein and of protein in absence of dye on a UV-2450 spectrophotometer (Shimadzu, Japan). Individual scattering contribution of IBs spectra was subtracted from their respective samples spectra (dye + protein). The final reaction mixture for thioflavin-T (Th-T) assay consisted of 50 µg/ml protein, 10 mM phosphate buffer of pH 7.0, 150 mM NaCl, and 75 µM Th-T. Samples were kept at room temperature for 10 minutes for thermal equilibration. Fluorescence emission spectra were recorded from 460 to 600 nm using an excitation wavelength of 440 nm (Cary Eclipse spectrophotometer, Varian, Australia). 5 nm slit width was fixed for both excitation and emission wavelength.

### ATR-FTIR spectroscopy of inclusion bodies

Asparaginase and hGH inclusion bodies were purified by sucrose density gradient method. Inclusion bodies were lyophilized to remove water. Samples were dried in a Speed-Vac system for 2 hours. The dried samples were spread on BaF2 crystal cells for spectra acquisition. The structure of dry IB aggregates was analyzed directly in Bruker Tensor FTIR spectrometer. For each spectrum (4000 cm^−1^ to 1000 cm^−1^), 25 interferograms were collected and averaged. Second derivatives of the amide I and II region spectra were used to determine the frequencies at which the different spectral components were located. These frequencies were used for assignment of secondary structural contents in inclusion body proteins.
